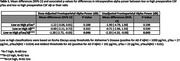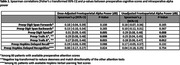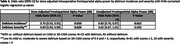# Anesthetic dose‐adjusted intraoperative alpha power in association with biomarkers of preclinical Alzheimer’s disease, preoperative attention function, and postoperative delirium

**DOI:** 10.1002/alz.089088

**Published:** 2025-01-09

**Authors:** Melody Reese, Mary Cooter, Kenneth C Roberts, Marty G. Woldorff, Jeffrey N. Browndyke, Leslie M. Shaw, Teresa Waligorska, Henrik Zetterberg, Kaj Blennow, Miles Berger, the MADCO‐PC, INTUIT, and MARBLE Investigators

**Affiliations:** ^1^ Duke University Medical Center, Durham, NC USA; ^2^ Duke Center for the Study of Aging and Human Development, Durham, NC USA; ^3^ Duke University, Durham, NC USA; ^4^ Duke Brain Imaging & Analysis Center, Durham, NC USA; ^5^ Duke Institute for Brain Sciences, Durham, NC USA; ^6^ Center for Neurodegenerative Disease Research, University of Pennsylvania, Philadelphia, PA USA; ^7^ Department of Pathology and Laboratory Medicine, Perelman School of Medicine, University of Pennsylvania, Philadelphia, PA USA; ^8^ University of Pennsylvania, Philadelphia, PA USA; ^9^ Dept of Pathology & Laboratory Medicine, University of Pennsylvania, Perelman School of Medicine, Philadelphia, PA USA; ^10^ Perelman School of Medicine, University of Pennsylvania, Philadelphia, PA USA; ^11^ University of Gothenburg, Mölndal, Gothenburg Sweden; ^12^ Institute of Neuroscience and Physiology, Sahlgrenska Academy at the University of Gothenburg, Göteborg Sweden; ^13^ University of Gothenburg, Mölndal Sweden; ^14^ Duke/UNC Alzheimer’s Disease Research Center, Duke University School of Medicine, Durham, NC USA; ^15^ Center for the Study of Aging & Human Development, Duke University, Durham, NC USA; ^16^ Duke University Center for Cognitive Neuroscience, Durham, NC USA

## Abstract

**Background:**

Postoperative delirium (POD) is characterized by fluctuating attention after surgery and is associated with increased risk of developing Alzheimer’s Disease (AD). While the neurophysiological changes that underlie POD and increased risk of AD are unclear, recent data has raised the possibility that an exaggerated brain response to anesthetics may be a biomarker for POD risk and preclinical AD‐like pathology. Thus, we examined whether anesthetic‐dose‐adjusted intraoperative brain activity is associated with POD or preoperative brain vulnerabilities (preclinical AD‐like pathology, preoperative inattention) that may contribute to risk of POD (and later AD).

**Method:**

82 patients from 3 prospective cohort studies of non‐cardiac, non‐neurologic surgical patients age ≥60 who had sufficient 32‐channel intraoperative electroencephalographic (EEG) data and received a gas anesthetic for ≥80% of their surgery were included. Other data included preoperative cerebrospinal fluid (CSF) samples, preoperative cognitive testing, and delirium scores (before surgery and twice daily for ≤5 days after surgery). Univariable associations were evaluated between 8‐12Hz anesthetic‐dose‐adjusted frontoparietal EEG alpha power and 1) preoperative CSF AD biomarkers (pTau181p, Aβ42, pTau181p/Aβ42), 2) preoperative attention function, and 3) POD incidence and severity. To account for the brain’s response to anesthetics, alpha power was dose‐adjusted as follows: (alpha power at each minute of surgery) / (2.5 – age‐adjusted minimum alveolar concentration at each minute of surgery) and summarized by patient across minutes as the mean of median‐filtered alpha.

**Result:**

Lower dose‐adjusted intraoperative frontoparietal alpha power was found among patients 1) with (vs. without) preoperative pTau/Aβ pathology (Table 1), and 2) among those who scored worse on timed preoperative attention tasks (Table 2). Low dose‐adjusted alpha power was also associated with increased odds of POD and moderate‐to‐severe delirium symptomology (Table 3). The conclusions were similar for unadjusted alpha power, though only dose‐adjusted values were associated with POD severity. Overall, the results suggest that, regardless of our anesthetic‐dose‐adjustment, low intraoperative frontoparietal alpha power is associated with CSF pTau/Aβ pathology, attention function, and POD.

**Conclusion:**

Future studies are needed to determine whether there are causal pathways between preoperative brain vulnerabilities, intraoperative frontoparietal alpha power, and POD. Understanding these pathways will improve our ability to identify at‐risk patients for targeted interventions.